# Bacterial Diversity and Interaction Networks of *Agave lechuguilla* Rhizosphere Differ Significantly From Bulk Soil in the Oligotrophic Basin of Cuatro Cienegas

**DOI:** 10.3389/fpls.2020.01028

**Published:** 2020-07-16

**Authors:** Nguyen E. López-Lozano, Andrea Echeverría Molinar, Elizabeth Alejandra Ortiz Durán, Maribel Hernández Rosales, Valeria Souza

**Affiliations:** ^1^ CONACyT-División de Ciencias Ambientales, Instituto Potosino de Investigación Científica y Tecnológica (IPICyT), San Luis Potosí, Mexico; ^2^ Centro de Física Aplicada y Tecnología Avanzada, Universidad Nacional Autónoma de México, Juriquilla, Mexico; ^3^ CONACyT-Instituto de Matemáticas, Universidad Nacional Autónoma de México, Juriquilla, Mexico; ^4^ Departamento de Ecología Evolutiva, Instituto de Ecología, Universidad Nacional Autónoma de México, Ciudad de México, Mexico

**Keywords:** agave microbiome, microbial co-occurrence, keystone species, community assembly, functional traits

## Abstract

Due to the environmental conditions presented in arid zones, it is expected to have a high influence of deterministic processes over the community assemblages. Symbiotic interactions with microorganisms could increase colonization and survival of plants in difficult conditions, independent of the plants physiological and morphological characteristics. In this context, the microbial communities associated to plants that inhabit these types of areas can be a good model to understand the community assembly processes. We investigated the influence of stochastic and deterministic processes in the assemblage of rhizosphere microbial communities of *Agave lechuguilla* and bulk soil on the Cuatro Cienegas Basin, a site known for its oligotrophic conditions. We hypothesize that rhizospheric microbial communities of *A. lechuguilla* differ from those of bulk soil as they differ in physicochemical properties of soil and biotic interactions, including not only the plant, but also their microbial co-occurrence networks, it is expected that microbial species usually critical for plant growth and health are more common in the rhizosphere, whereas in the bulk soil microbial species related to the resistance to abiotic stress are more abundant. In order to confirm this hypothesis, 16S rRNA gene was sequenced by Illumina from rhizospheric and bulk soil samples in two seasons, also the physicochemical properties of the soil were determined. Our results showed differences in bacterial diversity, community composition, potential functions, and interaction networks between the rhizosphere samples and the ones from bulk soil. Although community structure arises from a complex interplay between deterministic and stochastic forces, our results suggest that *A. lechuguilla* recruits specific rhizospheric microbes with functional traits that benefits the plant through growth promotion and nutrition. This selection follows principally a deterministic process that shapes the rhizospheric microbial communities, directed by the plant modifications around the roots but also subjected to the influence of other environmental variables, such as seasonality and soil properties. Interestingly, keystone taxa in the interactions networks, not necessarily belong to the most abundant taxonomic groups, but they have an important role by their functional traits and keeping the connections on the community network.

## Introduction

Soil microbes represent most of the biodiversity in terrestrial ecosystems and are main contributors to the preservation of soil quality and functioning (Philippot et al., 2013). Within the soil system, the rhizosphere, defined as “the immediate surroundings of the plant root”, is a highly dynamic interface, rich in microbial diversity and biomass (Philippot et al., 2013). The rhizosphere harbors complex microbial communities, whose dynamic associations are critical for plant growth and health, since many microorganisms increase nutrient availability (Richardson et al., 2009), contribute to the plant hormonal balance (Spaepen et al., 2007), prevent the attack of plant pathogens by antibiotic and antifungal compounds (Saharan and Nehra, 2011; Doornbos et al., 2012), to give some examples. Interestingly, these relationships are context-dependent, that under some conditions usually mutualistic taxa can become parasitic (Philippot et al., 2013). These complex interactions are mediated by plant-released nutrients, which act as one of the main regulators of microbial diversity, abundance, and activity in the rhizosphere (Sasse et al., 2018; Zhalnina et al., 2018). Despite part of the plant microbiome is determined by vertically transmitted endophytes (Shahzad et al., 2018), the bulk soil, adjacent root-free soil, is the principal source of the species richness in the rhizosphere. By comparing differences in taxonomic and functional traits between rhizosphere and bulk soil communities, we can get insights into how the selection processes operating in the rhizosphere are based in functionality (Mendes et al., 2014; Yan et al., 2017); or there are other factors involved. Although the composition of rhizosphere bacterial assemblages has been extensively studied (Compant et al., 2019), identifying and defining the interactions that occur among soil microorganisms has been little explored, despite its importance to understand microbial diversity and function (Shi et al., 2016).

Several hypotheses have been raised regarding microbial community assembly, it has been proposed that a combination of deterministic and stochastic processes shape microbial communities (Nemergut et al., 2013). Some examples of stochastic processes that produce random patterns in species co-occurrence are dispersal limitation, mass effects, and random demographics (Martiny et al., 2006; Vellend, 2010). In contrast, deterministic processes, are driven by niche partitioning and species interactions, producing segregation or even aggregation of the species (for example in the rhizosphere). The effect of deterministic processes is more notorious when extreme changes in crucial abiotic variables occur, this is the case of such as seasonal variation or changes in soil physicochemical properties (Chave, 2004; Dornelas et al., 2006; Chase, 2007). Due to the environmental conditions presented in arid zones, deterministic processes are expected to be very influential given their natural fluctuations and constant stresses. Arid zones are characterized by a frequent hydric stress, low organic matter, and nutrient content, particularly of nitrogen and phosphorus, as well as high salinity, temperatures, and exposure to UV radiation. Nevertheless, plants living in these habitats are well adapted to survive difficult conditions but independently due to their physiologic and morphological adaptations. It has been suggested that, symbiotic interactions with microorganisms could increase colonization and survival of plants in difficult conditions, independent of the plants physiological and morphological characteristics (Bashan et al., 1995; Puente et al., 2009). In this context, the microbial communities associated to plants that inhabit these arid soils can be a good model to understand assembly processes. *Agave lechuguilla*, is a very common plant in CCB as it is part of the desert scrub in arid and semiarid zones, having the widest natural distribution comparing it with the other Agave species (Gentry, 1982). Usually *A. lechuguilla* is found in rocky soils of limestone origin, it is considered ecologically important because is associated with soil formation and stabilization, and it has been reported as nurse plant for some cactus species (Silva-Montellano and Eguiarte, 2003). In arid rural areas of Mexico, it has economic importance (Castillo Quiroz et al., 2013) as well as potential biotechnological applications (Morales-Luckie et al., 2016). However, its microbial diversity has not been explored yet.

It has been demonstrated that plants in arid ecosystems, including some *Agave* species, are able to reconditioning, their surrounding soil, driving the selection and recruitment of associated microbes in the rhizosphere. This process, generally results in a significantly difference in the diversity, as well as in the microbial interaction networks, among rhizosphere and bulk soil (Desgarennes et al., 2014; Coleman-Derr et al., 2016; Marasco et al., 2018; Mosqueira et al., 2019). One of the factors that can drastically affect microbial communities in arid ecosystems are the climatic variations derived from seasonality. Interestingly, for some *Agave* species only the endophytic microbial communities have shown differences between seasons, but not the rhizospheric microbial communities (Coleman-Derr et al., 2016). However, the *Agave* species analyzed in the literature, generally grow as independent individuals exposed to the sun, and therefore, to evaporation. In contrast, *A. lechuguilla* forms patches of various individuals, which could allow a higher concentration of exudates and humidity, we believe, that, as a consequence, their rhizospheric effect could be enhanced. In this scenery, rhizospheric conditions could be more favorable for microbial communities having a greater amount of C sources and humidity that ameliorate the hard conditions during dry season, in contrast bulk soil is more susceptible to desiccation and high temperatures.

The Cuatro Ciénegas Basin (CCB) is located in the Chihuahuan Desert of Mexico. This place has an arid climate with two outstanding seasons: dry season from November to April and rainy season from May to October. It is geographically isolated and presents highly spatial heterogeneity because of its topology and irregular availability of water (Meyer, 1973). In addition to this, it is characterized by haline and oligotrophic soils (López-Lozano et al., 2012; Tapia-Torres et al., 2015; Pajares et al., 2016). These features caused an enormous diversification and a considerable amount of endemism, being reported as the place with the greatest number of endemism in North America, including macroorganisms and microorganisms (Souza et al., 2006; Alcaraz et al., 2008; Cerritos et al., 2008; Desnues et al., 2008; Escalante et al., 2009). *A. lechuguilla*, is a very common plant in CCB as it is part of the desert scrub in arid and semiarid zones, having the widest natural distribution comparing it with the other *Agave* species (Gentry, 1982). Usually, *A. lechuguilla* is found in rocky soils of limestone origin, it is considered ecologically important because it is associated with soil formation and stabilization, and it has been reported as nurse plant for some cactus species (Silva-Montellano and Eguiarte, 2003 *)*. In arid rural areas of Mexico, it has economic importance (Castillo Quiroz et al., 2013) as well as potential biotechnological applications (Morales-Luckie et al., 2016). However, *A. lechuguilla* associated microbial diversity has not been explored yet.

According with the aforementioned ideas, we investigated the influence of stochastic and deterministic processes in the assemblage of rhizospheric microbial communities of *A. lechuguilla* and bulk soil on the Cuatro Cienegas Basin. We hypothesized that rhizospheric microbial communities of *A. lechuguilla* differ from those of bulk soil as they differ in physicochemical properties of soil and biotic interactions. These effects will include not only the plant, but also their microbial co-occurrence networks. Under this scenario, it is expected that microbial species usually critical for plant growth and health are more common in the rhizosphere, whereas in the bulk soil microbial species related to the resistance to abiotic stress are more abundant.

## Materials And Methods

### Sample Collection

Four sites at the Cuatro Cienegas Basin (CCB) were selected by the presence of *A. lechuguilla* populations: “Becerra” — 26° 52.758′ N, 102° 08.19′W; “Carranza” — 26°59.519′ N, 102° 02.741′ W, “Orozco” — 26° 54.478′ N, 102° 07.169′ W, and “Madera” — 26° 57.609′ N, 102° 10.523′ W (**Figure S1**). The vegetation in the four sites was xerophytic scrub dominated by individuals of *A. lechuguilla, Larrea tridentata,* and some cacti species (**Figures S4A, B**). In each site, a sampling unit of 8 m × 8 m was placed, inside three independent adult individuals of *A. lechuguilla* with at least 30 leaves and without signs of flowering (Silva-Montellano and Eguiarte, 2003), and three vegetation-free interspaces (bulk soil) were selected randomly (**Figures S4C, D**). Two samplings from the rhizosphere of *A. lechuguilla* and bulk soil at a depth of 10 cm were carried out, one in the dry season (March) and the other one in the rainy season (October) of 2016. The rhizosphere is very difficult to delimit by definition (Philippot et al., 2013), for this reason we considered the soil attached to the roots for molecular analyzes and also used the soil surrounding the roots for physicochemical analysis. For this sampling, four equidistant points were located around the root system for a better representation of the rhizosphere, it was dug to a depth of 10 cm looking for the roots, then sub-samples were taken from the fraction that remains attached to the roots with a sterile spatula. The sub-samples were homogenized, and approximately 500 mg of soil were taken and stored in 2-ml Eppendorf tubes, 1 ml of DNA/RNA Shield™ was added for the nucleic acid preservation. The samples were kept on ice until their transportation to the laboratory and then stored at −80°C until its analysis. At the same time, we took as much soil as possible that was adhered to the roots, as well as the soil surrounding them was collected (approximately 500 g), to characterize the soil physicochemical properties, this sample was homogenized and was considered also as “rhizospheric soil.” These soil samples were kept in dark plastic bags, sealed, and stored at 4°C until its posterior analysis. As a result, in total 48 samples were collected, both for the microbial community analysis and soil physicochemical properties characterization.

### Soil Physicochemical Properties Analyses

Soil samples were oven-dried at 60°C, after that water content was determined using the gravimetric method. Soil pH and electric conductivity (EC) were measured in deionized water (soil/solution ratio, 1:2 w/v). Organic matter (OM) was determined by the calcination method (Schulte and Hopkins, 1996). Total carbon (TC) and total nitrogen (TN) were quantified with an elemental combustion system (4010, Costech Analytical Technologies, Valencia, CA). To measure soluble Phosphorous (P) and cations (Na+, K+, Ca2+, Mg2+) an inductively coupled plasma optical emission spectroscopy was used (ICP-OES) (730-ES, Varian, Palo Alto, CA). Finally, Ammonium (NH4+) and nitrate (NO3-) concentrations were determined using colorimetric methods (Forster, 1995; Miranda et al., 2001; Doane and Horwáth, 2003).

### Characterization of Microbial Communities

DNA was extracted using ZR Soil Microbe Miniprep™ kit (Zymo Research, Irvine, CA) following manufacturer's protocol, using 350 mg of soil. For the microbe characterization, the V3-V4 regions of the 16S rRNA gen were amplified using the primers 357 forward (5′-CTCCTACGGGAGGCAGCAG-3′) and CD reverse (5′-CTTGTGCGGGCCCCCGTCAATTC-3′). Illumina Miseq (Illumina, San Diego, CA) method was used for sequencing in pair-end 2 bp × 300 bp format at the LANGEBIO (Laboratorio Nacional de Genómica para la Biodiversidad).

The reads were assembled and filtered according to their quality (value of Q = 25, length = 300 bp, elimination of primers, barcodes, chimeras, and homopolymers≥ 8) using Mothur 1.36.1 (Schloss et al., 2009). Chimeras were identified using VSEARCH algorithm (Rognes et al., 2016) and removed. Sequences were classified using SILVA 123 (www.arb-silva.de) reference data base. The sequences that were classified as chloroplast, mitochondria, archaea or unassigned were removed as well as the singletons. In addition, to determine the diversity of the microbial communities, OTUs with a 97% of similarity were generated to calculate Chao1 estimator and Shannon index. A total of 6,837,394 reads were analyzed, after quality filtering, an average of 142,445 sequences were obtained for each library (**Table S1**). To avoid the bias of different depths of sequencing in the alpha and beta diversity measurements, the libraries were normalized to the same size (60,618 sequences) based on the library with the lowest number of sequences.

Based on literature review we identify possible changes in the relative abundance of functional guilds between soil types and seasons, using only the genera that were classified taxonomically with a degree of certainty greater than or equal to 80% of bootstrap according to the Naïve Bayesian Classifier (Wang et al., 2007). The genera classified as candidates or for which there is no information available in the literature constituted ~5% of the total community. We grouped the taxa into categories according to the presence of metabolic pathways that participate in the cycling of nutrients (considering the possession of genes involved in the trait or detected activity under laboratory conditions), its ability to tolerate stress or adapt to the environment (**Table S7**). The relative abundance of each functional category was calculated by adding the number of sequences of the genera within that category. A comparison between the treatments was made using ANOVA.

### Statistical Analyses

Shapiro-Wilk test was used to test the normality of the data. In case it does not have a normal distribution, data transformation was applied. Auto-correlation analysis of the physicochemical properties of the soil, was performed using Pearson method. However, none of the properties presented a significant auto-correlation. Afterwards, to look for differences between soil type (rhizosphere or bulk soil) and season, as well as the interaction between both variables, a two-way ANOVA was used. To verify the model fit, the normality assumptions of the residuals were assessed. As post-hoc evaluation for those properties presenting significant differences, Tukey test was applied. These analyses were carried out with using R software v 3.4.0., which was used in all posterior analyses. Ordination analysis was carried out using Non-Metric Multidimensional Scaling (NMDS) method based on Bray Curtis dissimilarity index, for this all analysis, only genera with relative abundance greater than 1% were considered. An environmental fit analysis of abiotic variables was applied (physicochemical properties of soil). These calculations were carried out with vegan package and the graphics with ggplot2 package. Heatmaps were constructed using gplots package, the clustering analyses on them were based in Bray Curtis dissimilarity index. PERMANOVA analyses was performed using the function adonis2 in vegan package.

### Interaction Networks

The inference of interaction networks was done using the software MetaMIS (Shaw et al., 2016) that uses a time-series Lotka-Volterra approach. Using the raw abundance data, we obtain the underlying interactions among the OTUs found in Agave rhizosphere and Bulk soil. The consensus networks inferred by MetaMIS are directed networks that include positive and negative interactions.

In order to analyze the interactions among the OTUs, we made some improvements to the software NetAn (https://github.com/valdeanda/NetAn) first used in (De Anda et al., 2018) and used it to obtain the global features of the interaction networks. NetAn is a network analysis tool programmed in Python, which extracts the following properties: order, size, density, diameter, radius, clustering coefficient, mean degree, centrality, hubs, vertex, and edge min-cut sets, connected components, cycles, maximal independent sets, degree distribution, modularity, communities. Some of these properties, such as diameter and radius, are calculated over the underlying undirected network.

We identified key features that show to be significant in comparison with random networks. NetAn generates hundred random networks with the same size and order of the ecological network, then extracts all the properties mentioned above and averages them. The values of each property were the compared to that of the ecological network, and we focus on the analysis of such features that are significant.

We also used Mfinder (Milo et al., 2002) to identify network motifs that can play an important role as building blocks of these networks, however, no network motif showed to be significant, telling us about the independency of the taxa in the network.

## Results

### Physicochemical Properties of Soils

ANOVA results indicated that water content and pH presented significant differences (P < 0.05) between Agave rhizosphere and bulk soil samples; Ca2+, NH4+, TN, and C/N ratio were different between seasons; NO3-, EC, OM, K+, Mg2+ and P were different in both conditions (season and soil type). Na+ and TC did not present significant differences in any condition (**Table 1**).

**Table 1:** Average and standard error values obtained from physicochemical properties of *Agave lechuguilla* rhizosphere and bulk soil in the interspaces.

Physicochemical property	Dry season		Rainy season		*F* value		
	Rhizosphere	Bulk soil	Rhizosphere	Bulk soil	Season	Soil type	Interaction
**Moisture (%)**	14.85 ± 3.12^a^	7.65 ± 2.41^b^	12.67 ± 2.82^ac^	11.04 ± 2.77^c^	0.643	30.643***	12.270**
**pH**	7.72 ± 0.31^a^	7.95 ± 0.34^ab^	7.87 ± 0.19^ab^	8.03 ± 0.19^b^	2.218	6.597*	0.205
**Ca^2+^ (mg/kg)**	1037.11 ± 78.90^a^	1004.68 ± 34.21^a^	1987.62 ± 148.62^b^	1917.81 ± 120.85^b^	945.317***	2.845	0.38
**NH_4_^+^ (mg/kg)**	3.04 ± 0.83^a^	2.06 ± 0.89^a^	13.55 ± 1.81^b^	14.10 ± 2.95^b^	454.684***	0.162	2.093
**TN (mg/kg)**	1.70 ± 0.49^a^	1.07 ± 0.43^a^	1.76 ± 1.02^a^	4.49 ± 2.67^b^	12.64***	1.644	18.491***
**C/N**	17.74 ± 7.72^a^	30.76 ± 2.61^a^	25.80 ± 6.50^a^	7.92 ± 2.30^b^	14.191***	2.316	15.935***
**NO_3_^-^ (mg/kg)**	25.95 ± 6.49^a^	18.21 ± 10.76^b^	49.48 ± 13.73^c^	28.81 ± 7.20^a^	33.075***	22.616***	1.408
**EC (μS/cm)**	322.31 ± 88.15^a^	224.34 ± 75.50^bc^	232.97 ± 47.57^b^	179.02 ± 20.07^c^	11.010**	18.968***	0.827
**OM (%)**	3.61 ± 1.69^ab^	1.98 ± 0.62^a^	3.98 ± 1.11^b^	2.94 ± 0.73^ab^	10.18**	21.09***	2.43
**K^+^ (mg/kg)**	186.22 ± 56.60^ac^	125.15 ± 42.49^a^	305.90 ± 81.11^b^	228.87 ± 49.43^c^	42.682***	16.309***	0.218
**Mg^2+^ (mg/kg)**	102.34 ± 34.14^ac^	70.09 ± 12.05^a^	164.83 ± 32.35^b^	134.34 ± 26.09^c^	63.449***	15.554***	0.012
**P (mg/kg)**	0.17 ± 0.09^a^	0.11 ± 0.05^a^	0.80 ± 0.12^b^	0.61 ± 0.09^c^	445.656***	20.865***	5.534*
**Na^+^ (mg/kg)**	6.79 ± 2.34^a^	6.56 ± 1.48^a^	8.44 ± 2.61^a^	7.13 ± 1.34^a^	5.71	0.731	0.699
**TC (mg/kg)**	27.48 ± 7.43^a^	29.48 ± 6.21^a^	26.35 ± 11.85^a^	21.54 ± 9.85^a^	2.975	0.286	1.677

Letters beside standard deviation indicate significant differences.

***p ≤ 0.001, **p ≤ 0.01, *p ≤ 0.05.

### Bacterial Composition and Diversity

Diversity Shannon-Wiener index presented significant differences (P < 0.05) between rhizosphere and bulk soil conditions, diversity was higher on the rhizosphere compared to bulk soil (6.88 ± 0.2 vs. 6.74 ± 0.2). There was not any significant difference between seasons. **Table S1** provides the data of observed OTUs, Chao1 estimator, Shannon-Wiener, and Simpson index values for every sample, and **Table S2** provides the results of the two-way ANOVA.

**Figure 1:**
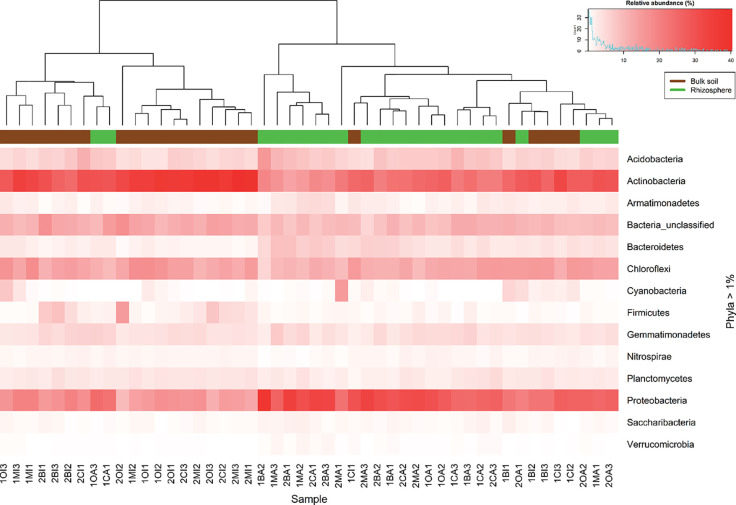
Heatmap constructed using the relative abundance of the identified phyla in all samples. The intensity of red color represents relative abundance. Superior dendogram is the result of the clustering analysis using Bray Curtis dissimilarity index, each sample is colored according to their origin, rhizosphere (green) or bulk soil (brown).

The most abundant phyla in all samples were Acidobacteria, Actinobacteria, Chloroflexi, and Proteobacteria. At this level, there was not so much variation on the relative abundance between seasons, except for Firmicutes which increase its relative abundance in rainy season (**Table S5**). Between rhizosphere and bulk soil Actinobacteria were more abundant in bulk soil, unlike the Proteobacteria which were more abundant in the rhizosphere (**Figure 1**, **Table S5**). The analysis at genus level of the most abundant phyla (considering only the genera with a relative abundance greater or equal to 1% in at least one sample, **Figure S2** and **Table S5**) revealed that *Rhizobiales FFCH5858,* and *Kallotenuales AKIW781* were significantly (P < 0.05) more abundant in the dry season, and *Actinobacteria TakashiACB11* and *Acidobacteria S6* increased its relative abundance in rainy season. According to the soil type, *Sphingomonas, Microvirga, Rhizobiales JG34KF361, Thermomicrobia JG30KFCM45* and *Bryobacter* increased their relative abundance in the rhizosphere; and *Chloroflexi TK10*, *Kallotenuales AKIW781*, *Actinobacteria TakashiACB11*, *Rubrobacter,* and *Actinobacteria MBA2108* had a preference for bulk soil (**Table S6**).

**Figure 2:**
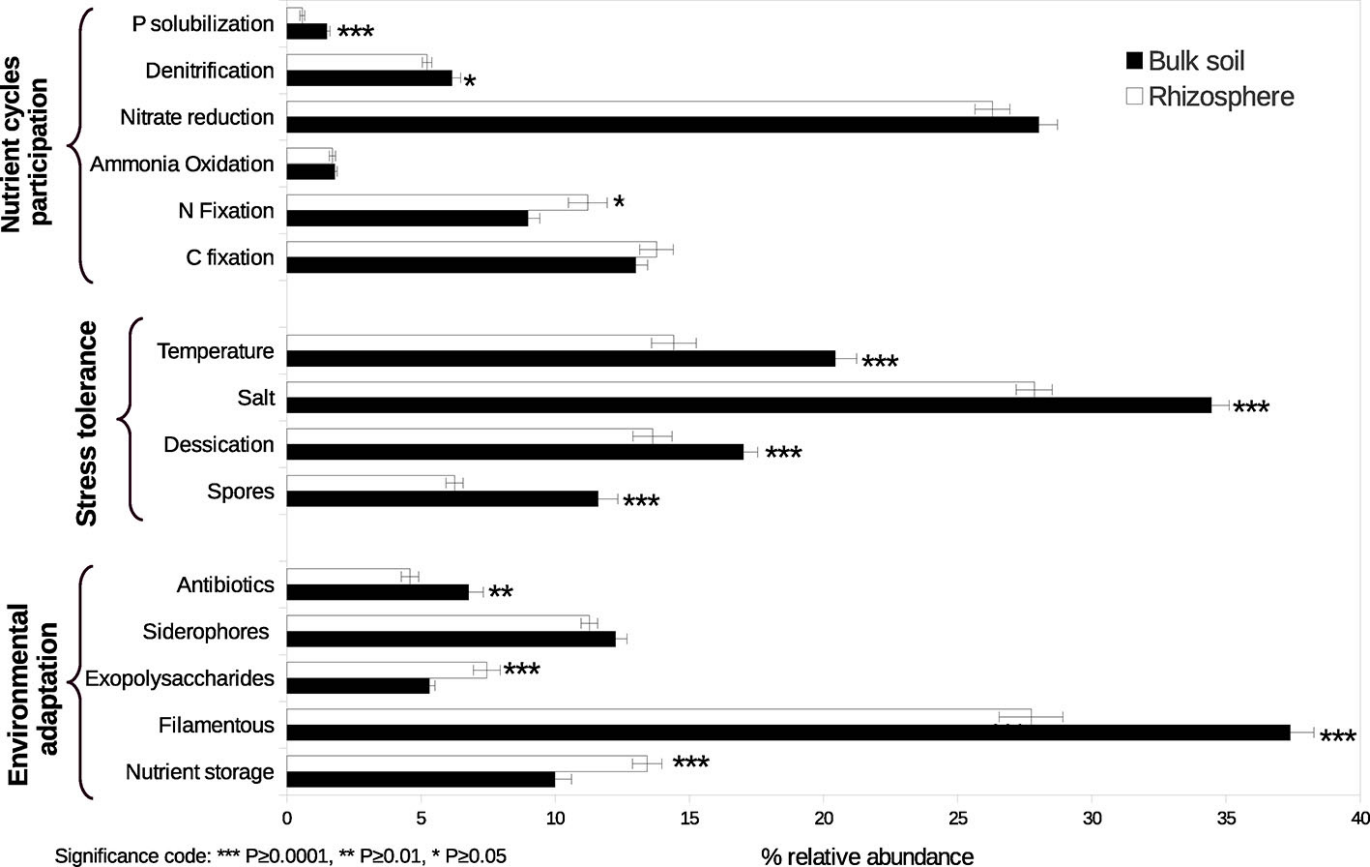
Relative abundance of functional guilds participating in nutrient cycles, traits conferring stress tolerance, and traits conferring environmental adaptation in rhizosphere and bulk soil. Asterisks indicate significant differences. Bars represent standard errors.

As a result of the analysis based on literature review about the functional capacities of the taxa identified, significant changes between conditions in the relative abundance of some categories were observed (**Figure 2**, **Table S4**). We found a higher relative abundance of N fixers in the rhizosphere of *A. lechuguilla* than in the bulk soil, represented principally by *Sphingomonadales* and *Rhizobiales*. In contrast, denitrifiers and P solubilizers were both more abundant in bulk soil. Particularly, denitrifiers were primarily represented by the genera *Bacillus, Streptomyces, Euzebya,* and *Bosea*, whereas P solubilizers were represented by members of the family *Gaiellaceae*. In general, the groups whose stress tolerance has been reported were more abundant in the bulk soil. Regarding traits of environmental adaptation, genera with the reported capacity of antibiotic production and filamentous formation were more abundant in the bulk soil, while genera that produce exopolysaccharides and the capacity of nutrients storage (formation of polyhydroxyalkanoates and polyphosphates inclusions) were more abundant in the rizosphere.

**Figure 3:**
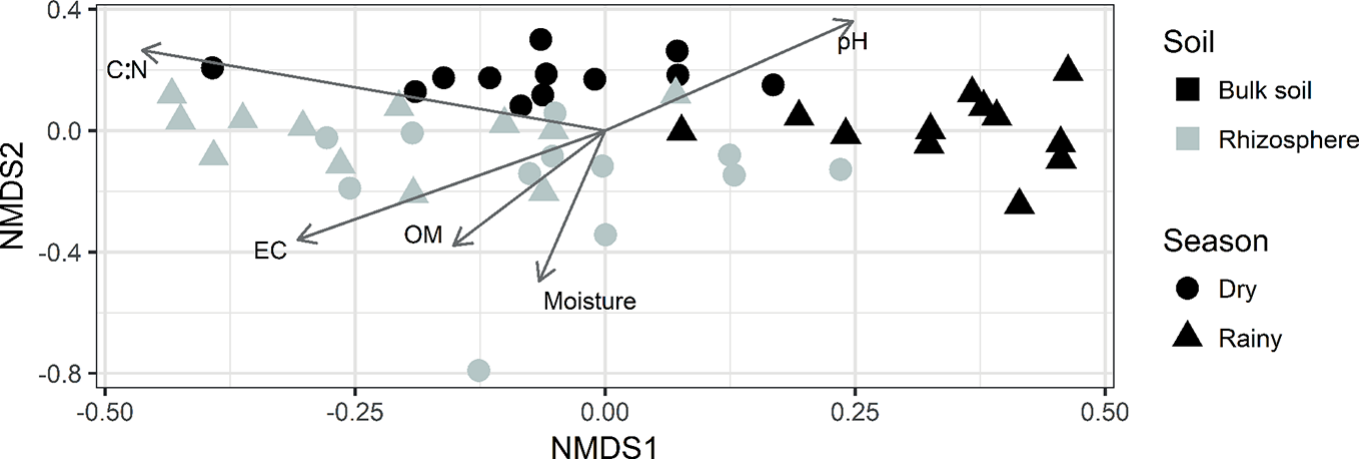
NMDS analysis using relative abundance at a genus level. Arrows indicate the physicochemical properties that resulted in a significant relationship with the microbial community.

The results of the NMDS analysis using the data from the bacterial composition at genus level (**Figure 3**) show that the most remarkable difference corresponds to the soil type and that the bulk soil community is more sensitive to variation caused by the season. Additionally, making the adjustment of physicochemical properties data is observed that pH, EC, soil moisture, OM, and C/N ratio are the properties that show a significant relationship with the composition of the microbial community.

### Interaction Between Bacteria

**Figure 4:**
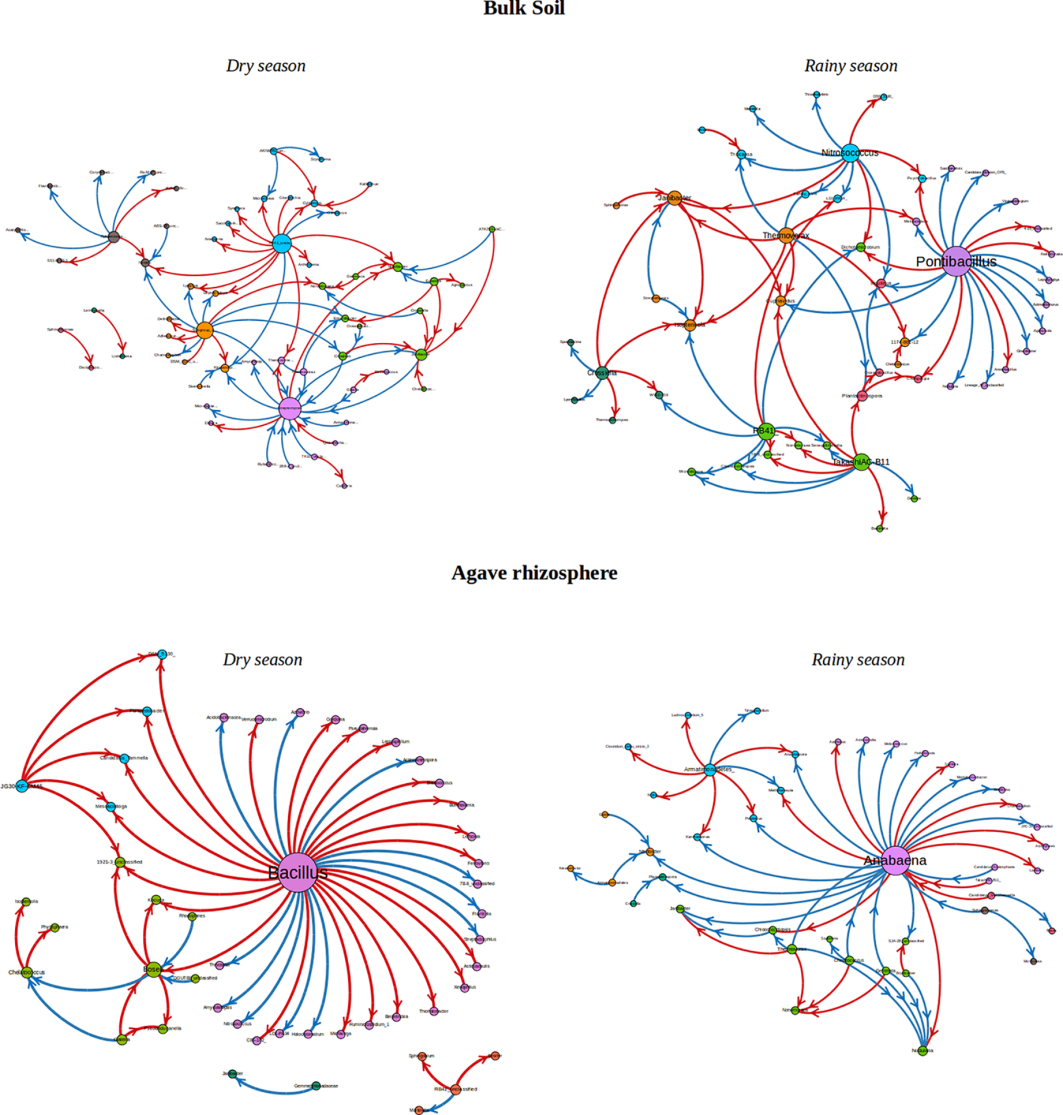
Interaction networks found for Rhizosphere and bulk soil over two seasons. The size of the nodes represents the importance in the network, i.e. the bigger the node is the more connections it has with other OTUs. Red rows are negative interactions, while blue rows are positive interactions. The color of each node represents the community to which it belongs.

To analyze the changes in the ecological interaction patterns between conditions, interactions networks were generated based in the relative abundance of the identified genera. In general, density and clustering coefficients of the networks were low, indicating that these communities have few strong interactions (**Table 2**, **Figure 4**), having a tendency to form few modules, even less than those formed in random networks. The interaction networks are not dense and show that the degree distribution follows a power law distribution, common to complex networks. Moreover, in these networks, there are just couple of nodes (and sometimes only one) that plays an important role, being the genera with more positive and negative interactions, which is very unlikely in random networks. The number of communities (modules) was between 5 and 8, and the number of nodes in each module varied between 3 and 15 (**Table S3**). The clustering coefficient was slightly higher in the rhizosphere than in the bulk soil networks when the data was separated by season.

**Table 2:** Global network measures obtained from real (consensus) and random networks.

	Both soils together		Rhizosphere			Bulk soil		
	Dry	Rainy	Both seasons together	Dry	Rainy	Both seasons together	Dry	Rainy
Total nodes	61	61	62	53	54	59	74	71
Density	0.017	0.022	0.020	0.023	0.030	0.021	0.021	0.029
Clustering Coefficient	0.000	0.016	0.023	0.039	0.037	0.028	0.012	0.018
Mean degree	1.017	1.151	1.017	1.104	1.256	1.093	1.233	1.420
Modularity	0.711	0.607	0.665	0.466	0.456	0.644	0.584	0.522
No. of modules	8	7	8	5	8	8	7	6

Interestingly, there was a high number of independent sets of genera that interact with the rest of the community through a single hub. The existence of these single hubs that connect a module with the rest of the network, could be due to the fact that OTUs within a genus were merged together. This is very interesting, since it indicates the importance of these genera in the community. In this sense, it would be very easy to split apart these networks by removing only one node or one connection (one genus), which tells us about the vulnerability of the network. If environment perturbations affect the network, it will be very likely that the interactions that maintain the dynamics of the network would be lost. It is important to note, that there are some networks which are disconnected, i.e. a network formed by a set of subnetworks, where only one of them contains most of the genera, and the others have only few members (from 2 to 7). In this study, we focused mainly in such subnetworks with 5 or more nodes. In each subnetwork is identified a connected component. Even though, most of the genera do not share interactions with other members in the community, as we can see by studying the independent sets, it is surprising that they are still able to form communities. Members of a community have more interactions among them, that with members outside the community. These communities are formed by a keystone hub and a set of genera which have no interactions among them. Since these important hubs could represent “key species” for the community structure, these strongly interconnected taxa or hubs in the networks were identified and the directionality of the interactions that they establish with the other members in the community was determined (**Figure 4**, **Table 3**). The hubs were different between type of soil and season. In the rhizosphere, *Saccharibacteria_unclassified, Bacillus,* and *Anabaena* were the genera with the highest number of interactions, being also the hubs with maximal out-degree (exert an effect on the other groups). *Bacillus* was the principal hub in the rhizosphere during dry season and Anabaena in the rainy season. Besides, *Ensifer, Bosea,* and *Anabaena* were the taxa with the maximal in-degree (the most affected by other members of the community). In the bulk soil, *Plantactinospora, Streptomyces,* and *Pontibacillus* were the genera with the highest number of interactions for all data, dry and rainy seasons, respectively. The hubs with the maximal out-degree were *Craurococcus, RB41_unclassified* and *Pontibacillus,* for all data, dry and rainy seasons, respectively. *Chroococcidiopsis* was the most affected by other members in the community for all data, while two genera (*Janibacter* and *Isoptericola*) and *Streptomyces* were the hubs with max-in degree in the dry and rainy seasons, respectively.

**Table 3:** Strongly interconnected taxa or hubs identified at genus level and their number of interactions in the community networks.

	Both soils		Rhizosphere			Bulk soil		
	Dry	Rainy	both seasons	Dry	Rainy	both seasons	Dry	Rainy
**Hubs with Max Total Degree**	*Janibacter, Bacillus*	*Anabaena*	*Saccharibacteria_unclassified, Bacillus*	*Bacillus*	*Anabaena*	*Plantactinospora*	*Streptomyces*	*Pontibacillus*
**Max Total Degree**	10	12	9	33	28	14	16	19
**Hubs with Max In Degree**	*Janibacter*	*Chroococcidiopsis, Asteroleplasma*	*Ensifer*	*Bosea*	*Anabaena*	*Chroococcidiopsis*	*Streptomyces*	*Janibacter, Isoptericola*
**Max In Degree**	8	8	5	4	7	7	10	5
**Hubs with Max Out Degree**	*Bacillus*	*Nonomuraea*	*Saccharibacteria_unclassified, Bacillus*	*Bacillus*	*Anabaena*	*Craurococcus*	*RB41_unclassified*	*Pontibacillus*
**Max Out Degree**	10	10	9	33	21	7	14	19

## Discussion

In the present investigation, the soil properties with the highest influence over the composition and structure of the microbial communities were pH, EC, moisture, OM, and C/N ratio. The soil in CC is alkaline with elevated concentrations of ions, resulting in high electrical conductivity. This characteristic can be attributed to the gypsum-rich nature of the CC soils. In previous studies, salinity has demonstrated a strong influence on the composition of the microbial communities in this Basin, not only because it is a selecting pressure by itself, but also because it decreases the availability of nutrients, which are very scarce in these soils (López-Lozano et al., 2012; Pajares et al., 2016). Coupled with this, the low nutrient availability to soil microbes and vegetation, observed in the low C/N ratio, suggests deficiency of soil organic C that can limit the N cycle. Despite the great heterogeneity of this arid environment, the oligotrophic conditions seem to be a general characteristic of the soils in all the Basin (López-Lozano et al., 2012; Tapia-Torres et al., 2015; Pajares et al., 2016).

The bacterial composition found in our samples clearly reflect their origin, since many of the identified taxa have been reported in arid zones (Walker and Pace, 2007; Bachar et al., 2010; Saul-Tcherkas and Steinberger, 2011; Andrew et al., 2012; López-Lozano et al., 2012; Marasco et al., 2012; Torres-Cortés et al., 2012; An et al., 2013; Kavamura et al., 2013; Desgarennes et al., 2014; Coleman-Derr et al., 2016; Postma et al., 2016). However, the presence of many bacterial groups can be related to the particular characteristics of CCB soils. For example, Acidobacteria (Fierer et al., 2007; Kielak et al., 2016), Armatimonadetes (Lee et al., 2014), Verrucomicrobia (Da Rocha et al., 2010; Senechkin et al., 2010; Bergmann et al., 2011), and Gemmatimonadetes (Zhou et al., 2007; Cockell et al., 2009) are commonly associated to oligotrophic places. In special, the phylum Gematimonadetes has been recognized for their high tolerance to desiccation and its ability to store phosphorus (DeBruyn et al., 2011).

Our results showed differences in bacterial diversity, community composition, potential functions, and interaction networks between the rhizosphere samples and the ones from the bulk soil, which indicate a rhizospheric effect. Higher Shannon index in the rhizosphere of other Agave species (*Agave salmiana, Agave tequilana,* and *Agave deserti*) than in bulk soil had been previously observed (Coleman-Derr et al., 2016), as well as differences in community composition (Desgarennes et al., 2014; Coleman-Derr et al., 2016). Even though these studies were focusing on differences between species and compartments of the plant (phyllosphere, endosphere, and rhizosphere), they found also significant differences between the microbial community of the zone near to the root and the microbial community of bulk soils. Despite *A. lechuguilla* is the smallest species within the genus Agave, this rizospheric effect was clearly observed. The plant presence improves the conditions for some groups of microorganisms; therefore, it changes the composition and structure of microbial communities in soil. Similar to our observations, some studies had found an increment in the relative abundance of Proteobacteria in the rhizosphere, showing a positive correlation with moisture (Marasco et al., 2012; Kavamura et al., 2013). While in bulk soil there is higher relative abundance of Actinobacteria, negatively correlated also with moisture (Saul-Tcherkas and Steinberger, 2011; Marasco et al., 2012; Kavamura et al., 2013). At genus level, for example *Bryobacter* of Acidobacteria phylum was more abundant in the rhizosphere, this might be because these microorganisms can obtain carbon from the decomposition of organic matter and nitrogen from nitrate (Kulichevskaya et al., 2010). Another example is *Mycobacterium*, which are found in sites rich in organic matter, humid, and even in outer layers of plant tissues (Kazda et al., 2010). Some of the identified bacteria are well known because they form associations with plants, like *Bradyrhizobium* and *Rhizobia* (Carareto et al., 2014; De Souza et al., 2014) and some others carry out nitrate to nitrite reduction like *Microvirga* (Kelly et al., 2014), *Craurococcus* (Saitoh et al., 1998) and *Microlunatus*; this last one has been reported as phosphorus accumulator too (Nakamura et al., 1995). On the contrary, other groups have adaptations that could explain their presence in higher proportions in the bulk soil of CC. For example, some members of Actinobacteria phylum are spore forming, have tolerance for dry conditions, high salinity, and high temperatures (Chen et al., 2004; Goodfellow et al., 2012). *Euzebya* needs salt to grow (Kurahashi et al., 2010), *Thermoleophilum,* and *Kallotenue* have tolerance to high temperatures as well (Zarilla and Perry, 1984; Cole et al., 2013). This last one group and *Ardenticatena* are also filamentous bacteria, characteristic that allows them to retain humidity and extend through the soil matrix to get more nutrients (Cole et al., 2013; Kawaichi et al., 2013).

Furthermore, analysis of the functional capacities of the taxa identified (**Table 2**), found a higher relative abundance of bacteria with the potential to fix N in the rhizosphere of *A. lechuguilla* than in bulk soil, represented principally by *Sphingomonadales* and *Rhizobiales.* Free-living N fixers that are associated with the rhizosphere of non-symbiotic plants can represent the principal source of new N in many ecosystems, due to the lack of large populations of plants in symbiosis with N fixers (Reed et al., 2011). It is well known that N fixation is a high energy-demanding process, for this reason the lower organic matter in bulk soil can be limiting the reducing power needed to fix N.

Other functional groups that showed differences in relative abundance between rhizosphere and bulk soil were P solubilizers and denitrifiers, both being more abundant in the bulk soil. P solubilizers were represented by two unknown genera of the family *Gaiellaceae*, a group in which some species possess alkaline and acid phosphatases (Albuquerque and da Costa, 2014). However, without a better description of isolated strains within these genera is not possible differentiate if their presence is due to habitat preference or to their functional capacities. With regard to denitrifiers, multiple factors have been shown to influence the activity and abundance of this diverse functional guild, including pH (Baggs et al., 2010), soil texture (Gu et al., 2013), organic matter (OM) (Barrett et al., 2016), and inorganic N (Niboyet et al., 2009). Our soil samples did not show significant differences in pH or texture, but had higher amounts of OM and nitrate in bulk soil. The higher relative abundance of denitrifiers observed in bulk soil may be due to a strong competition for nitrate between plants and denitrifiers in the rhizosphere.

In addition to the effect of the plant and physicochemical properties, other studies reported changes in the composition of soil microbial communities between seasons (Aguilera et al., 1999; Saul-Tcherkas and Steinberger, 2011; Lançoni et al., 2013). In this research, although the season factor was not significant for all the samples in the environmental fit perform over the NMDS analysis, in **Figure 3**, a differentiation between dry and rainy season was observed for the samples belonging to the bulk soil, this could be because the conditions in *Agave* rhizosphere remained more stable, avoiding the effect of the season over microbial communities. In a previous study in *Agave* plants, the influence of the season was only found in the microbial communities from the endosphere but not in the rhizosphere (Coleman-Derr et al., 2016). Despite the fact that soil samples used for physicochemical analyzes, came from a larger fraction of rhizospheric soil, than those taken for the molecular analyzes (which were closer to the root, hence more influenced by the plant). In this study, it was possible to observe a clear differentiation between both types of soil and seasons, as well as significant correlations between these properties and the relative abundance of some bacterial groups.

Our data show that soil type (rhizosphere or bulk soil) selects differentially bacterial traits, suggesting that abiotic filtering plays a significant role in this kind of ecosystems affected by hostile conditions (high soil electrical conductivity, water-limitation, low nutrients availability, etc.) (Goberna et al., 2014). The bulk soil exposed to high temperature fluctuations and radiation had more bacterial traits associated with tolerance to environmental stress, filtering bacteria tolerant to temperature, desiccation, salt, and with the capacity to form resistant structures (exospores, other spores, and cysts). In contrast, the “high” productive environment created in the *Agave* rhizosphere facilitates the nutrient storage and the exopolysaccharides production as competitive abilities (Goberna et al., 2014).

Our networks analysis reveals that interactions among bacterial genera followed a power-law distribution, this structure is widespread in many real-world (Internet, social, and biological) networks (Adamic and Huberman, 2000). Interestingly, we found that networks presented low complexity, characteristic that can be attributed to a dynamic community, highly variable by season, with a great number of taxa with no common co-occurrence across sites. This behavior can be the result of the great heterogeneity of the soils in the Basin, raveling the influence of stochastic processes in the assembly of these communities, another possibility is that our sampling size was too coarse to identify micro-scale covariations. However, at the scale of our sampling, the interactions detected must be strongly preserved to be distinguished. The identified modules within the networks are likely result of interactions or covariations between bacterial genera in response to shared niches in the rhizosphere and bulk soil. Topologically, we also identified network hubs and connectors, because they can function as keystone taxa that maintain the network structure (Faust and Raes, 2012). These keystone taxa are relatively more important than other taxa in the network, their loss may cause modules and networks to disassemble (Jordán et al., 2008), and thus their presence can be transcendental to maintain ecosystem stability (Sole and Montoya, 2001).

In the rhizosphere, the hubs most affected by other members of the community are involved in nutrient acquisition or enhance bioavailability of soil nutrients, *Bosea* and *Ensifer* are well-known N fixers and denitrifiers (Shinwari et al., 2019) and *Anabaena* is a filamentous Cyanobacteria with the capacity to fix N (Manjunath et al., 2016). We hypothesize that they could be subject to predation. Besides, the hubs that affect other members of the community in the rhizosphere were identified as *Saccharibacteria_unclassified, Bacillus,* and *Anabaena*. The phylum candidatus Saccharibacteria was formerly known as Candidate Division TM7 (Kindaichi et al., 2016). There are very few strains of *Saccharibacteria* isolated and characterized, for this reason, the information about their potential functions is limited. However, there are evidence that *Saccharibacteria* is related to wilt disease suppression (Zhang et al., 2016; Shen et al., 2018), but how it is involved in potential disease control remains unknown. Moreover, *Bacillus* spp. is considered to be safe microorganisms for the plants, having potent plant growth promoting traits such as IAA (indol acetic acid) production, nitrogen fixation, phosphate solubilization, and biocontrol attributes like production of hydrolytic enzymes, HCN (cyanogen), siderophores, and antibiotics (Kumar et al., 2012). Besides, *Anabaena* and other Cyanobacteria has been used in agriculture to improve the soil quality for their beneficial effects on plant health and productivity, since they produce diverse metabolites such as polysaccharides, betaines, micronutrients, and plant growth hormones (cytokinins, auxins, abscisic, and gibberellic acid). In specific, *Anabaena* extracts improve plant resistance to both biotic and abiotic stresses, including antimicrobial activity against different pathogens (Righini and Roberti, 2019). The importance of *Anabaena* in the community is enhanced during the rainy season, it has been reported that when the water content of alkali soils increases the biomass was dominated by *Anabaena* among others Cyanobacteria (Rao and Burns, 1991).

In contrast, some hubs in the bulk soil were members of Actinobacteria phylum (*Plantactinospora, Streptomyces, Janibacter*, and *Isoptericola*). Actinobacteria have a ubiquitous distribution in the biosphere being a dominant taxon in soil microbial communities (Bull, 2011). They have particular tolerance to high salinity and desiccation and have been isolated from many arid and hyper-arid deserts, including habitats considered as potential analogs of Mars (Stevenson and Hallsworth, 2014). Another characteristic of Actinobacteria is the large arsenal of secondary metabolites, nowadays about two-thirds of all naturally derived antibiotics in current clinical use, as well as antifungal, antihelmintic, and many anticancer compounds are produced by them. However, the few genomes of Actinobacteria strains isolated or recovered from environmental metagenomic data in arid environments suggest that there is a plenteous actinobacterial bioactive chemicals to be discovered (Thumar et al., 2010; Mohammadipanah and Wink, 2016).

Other interesting hubs were *Chroococcidiopsis* and *Craurococcus*, both aerobic photosynthetic bacteria which could play an important role as primary producers in the bulk soil. *Chroococcidiopsis* are Cyanobacteria extremely resistant to desiccation and ionizing radiation (Verseux et al., 2017), during nutrient deprivation cell divisions occurred and were able to survive after one moth of starvation (Billi and Grilli Caiola, 1996). *Craurococcus* are strictly aerobic and chemoorganotrophic, which produced Chla and carotenoids only under aerobic growth conditions, these genera was isolated from mesic soils has only one species described (Saitoh et al., 1998).

Finally, the hubs with the maximum out degree were *Pontibacillus* and *RB41 unclassified*. *Pontibacillus* has been described as a salt-tolerant microbe, with the capacity to fix nitrogen; solubilize zinc, potassium, and phosphorus; produce ammonia, HCN, siderophores and other secondary metabolites (Yadav and Saxena, 2018). *Actinobacteria RB41* from soils under low-nutrient or stress conditions was shown to be important in maintaining biogeochemical and metabolic functions (Foesel et al., 2013), since they are positive correlated with nitrogen and sulfur cycling, such as nitrification, sulfide oxidation, sulfite reduction, and dimethylsulfoniopropionate degradation (Wang et al., 2019). Interestingly, all these keystone taxa not necessarily belong to the most abundant groups. These suggest that relatively rare groups in the community have an important role by their functional traits and keeping the connections on the community network.

Although community structure arises from a complex interplay between deterministic and stochastic forces, our results suggest that *A. lechuguilla* recruits specific rhizospheric microbes with functional traits that benefits the plant through growth promotion, nutrition, and control disease. This selection follows principally a deterministic processes that shapes the rizhospheric microbial communities, directed by the plant modifications around the roots (Nicolitch et al., 2017; Fitzpatrick et al., 2018) but also subjected to the influence of other environmental variables, such as seasonality and soil properties. Further analyses are needed to better understand the mechanisms by which the plants inhabiting arid lands select their rhizospheric community, for example there is few information about the role of exudates secreted by CAM (crassulaceae acid metabolism) plants in shaping microbial communities. Furthermore, knowledge of factors involved in plant–microorganism interactions in oligotrophic and saline soils should be helpful for conservation efforts and reforestation projects that pretend to use native plants in this kind of areas.

## Data Availability Statement

The datasets generated for this study can be found in the https://www.ncbi.nlm.nih.gov/bioproject/?term=PRJNA553176.

## Author Contributions

This study was designed and coordinated by NL-L and VS. AE and NL-L performed the data collection, general analysis and interpretation. EO and MH performed the network analysis. NL-L and AE drafting the article. MH and VS performed a critical revision of the article. All authors contributed to the article and approved the submitted version.

## Funding

The authors acknowledge the funding of WWF-Alianza Carlos Slim, and the support by the SEP-CONACYT Basic Science 254406 to NL-L. AE thanks to CONACyT for the scholarship 588371.

## Conflict of Interest

The authors declare that the research was conducted in the absence of any commercial or financial relationships that could be construed as a potential conflict of interest.
